# Supplementary material for: Active expiration reduces hypercapnia in lung failure – results of the prospective interventional ActiveEx study and development of a prototype device for automated application
Source: PLoS One. 2025 Oct 16;20(10):e0333579. doi: 10.1371/journal.pone.0333579 (PMC12530571; doi:10.1371/journal.pone.0333579)

S1 Fig. Flowchart on data collection and execution of the study. This figure illustrates the process of data collection and the execution timeline and methodology of the study.

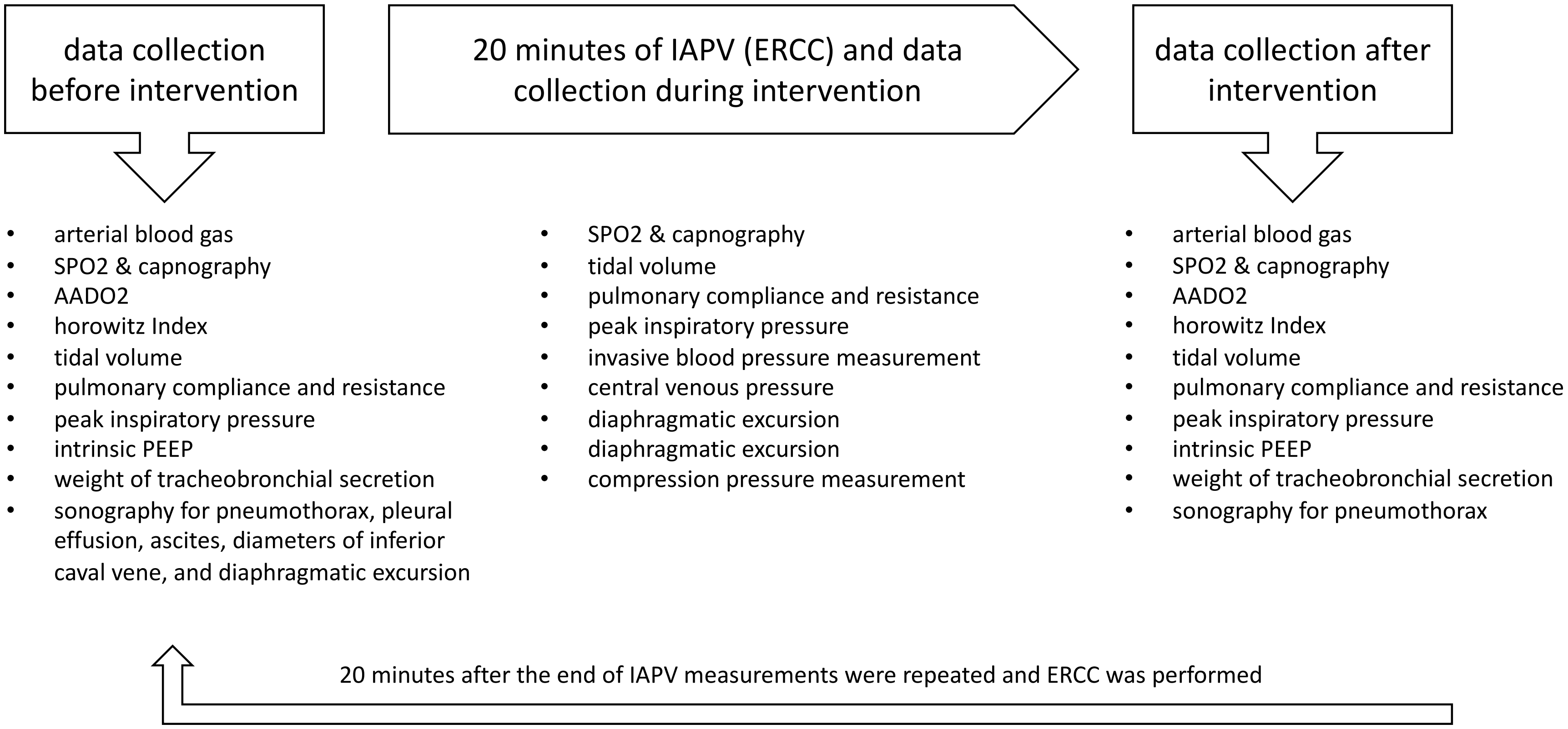

Supplement: S1 Fig — This figure illustrates the process of data collection and the execution timeline and methodology of the study. (PDF) [file pone.0333579.s001.pdf]
