# Supplementary material for: Active expiration reduces hypercapnia in lung failure – results of the prospective interventional ActiveEx study and development of a prototype device for automated application
Source: PLoS One. 2025 Oct 16;20(10):e0333579. doi: 10.1371/journal.pone.0333579 (PMC12530571; doi:10.1371/journal.pone.0333579)

S2 Fig. Air-filled cushion used for manual pressure measurement. Self-constructed manual pressure sensor used during IAPV (one sensor) or ERCC (two sensors)

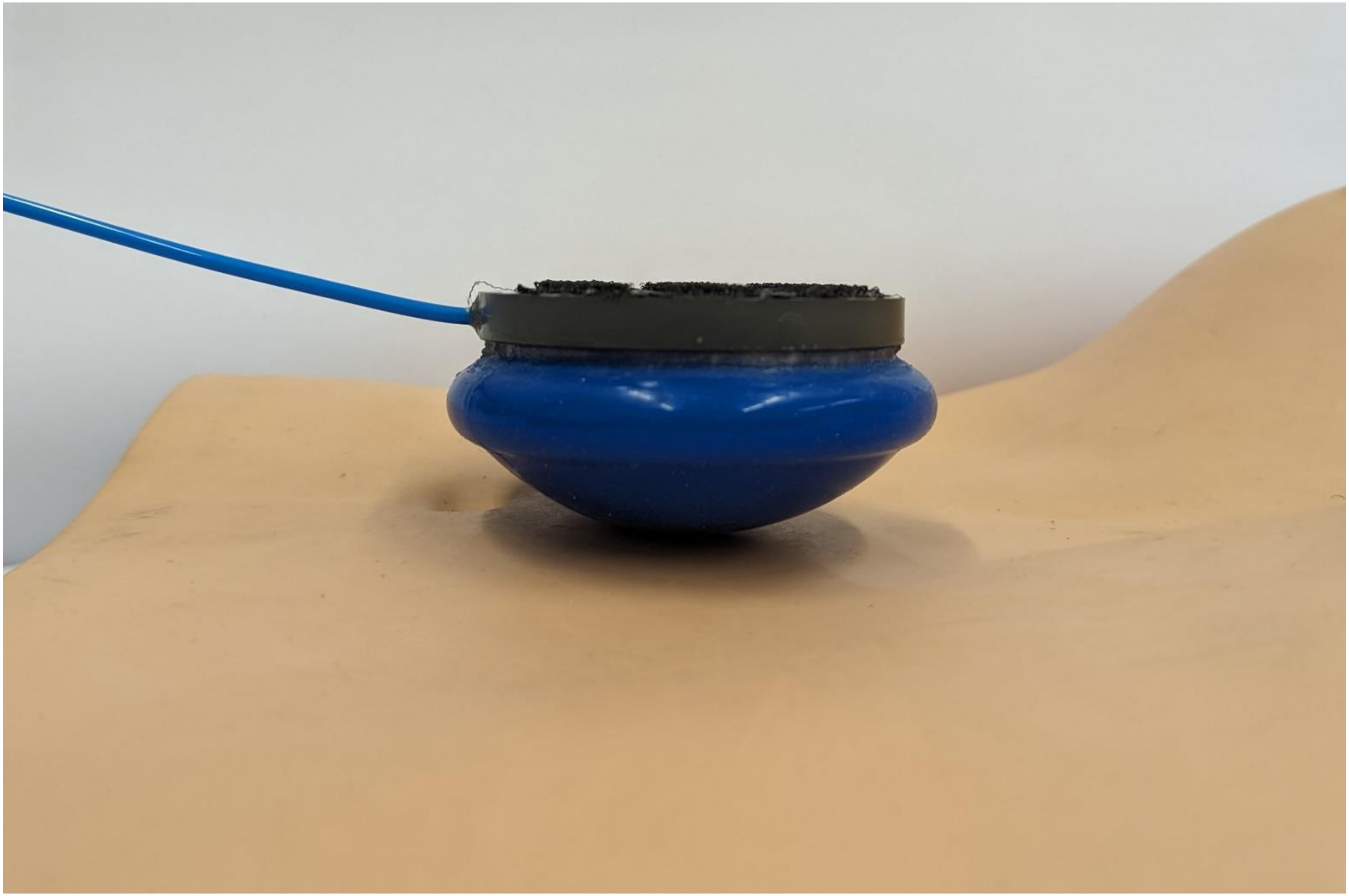

Supplement: S2 Fig — Self-constructed manual pressure sensor used during IAPV (one sensor) or ERCC (two sensors). (PDF) [file pone.0333579.s002.pdf]
