# Supplementary material for: Active expiration reduces hypercapnia in lung failure – results of the prospective interventional ActiveEx study and development of a prototype device for automated application
Source: PLoS One. 2025 Oct 16;20(10):e0333579. doi: 10.1371/journal.pone.0333579 (PMC12530571; doi:10.1371/journal.pone.0333579)
Supplement: S3 Fig — This figure presents images of lung ventilation patterns as recorded by the PulmoVista system. (PDF) [file pone.0333579.s003.pdf]

S3 Fig. Visualization of pulmonary ventilation by PulmoVista. This figure presents images of lung ventilation patterns as recorded by the PulmoVista system.

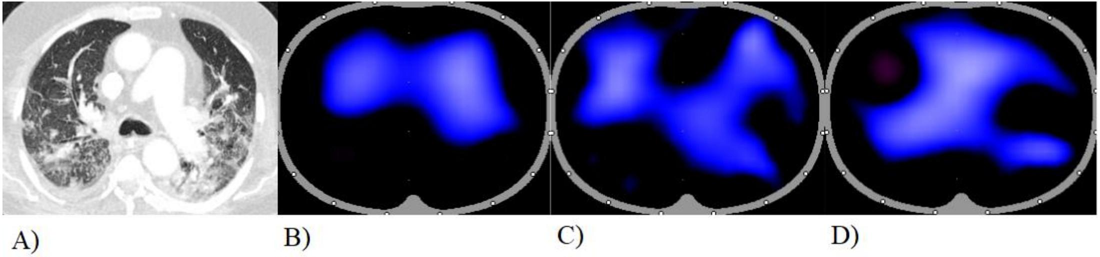

A) displays the native CT of the patient. B) Shows ventilated pulmonary areas before the study. C) Ventilation during IAPV. D) Ventilated areas during ERCC.
