# Supplementary material for: Active expiration reduces hypercapnia in lung failure – results of the prospective interventional ActiveEx study and development of a prototype device for automated application
Source: PLoS One. 2025 Oct 16;20(10):e0333579. doi: 10.1371/journal.pone.0333579 (PMC12530571; doi:10.1371/journal.pone.0333579)
Supplement: S1 Table — This table details individual patient tidal volumes and pressure settings pre- and during intervention. (DOCX) [file pone.0333579.s004.docx]

| **Patient** | **1** | **2** | **3**, | **4** | **5** | **6** | **7** | **8** | **9** |
| --- | --- | --- | --- | --- | --- | --- | --- | --- | --- |
| Baseline^1^ tidalvolume^2^ | 6.08  (5.94, 6.13) | 4.73  (4.72, 4.88) | 7.00  (6.95, 7.13) | 9.52  (9.45, 9.61) | 6.66  (6.54, 6.72) | 6.19  (5.71, 6.86) | 5.86  (5.80, 5.99) | 5.09  (4.74, 5.46) | 6.67  (6.53, 6.82) |
| IAPV^1^ tidalvolume^2^ | 9.33  (8.96, 9.64) | 9.59  (8.53, 9.99) | 8.61  (8.41, 8.79) | 11.51  (10.76, 13.20) | 14.19  (12.78, 16.23) | 8.00  (7.65, 8.24) | 6.99  (6.81, 7.40) | 7.63  (7.33, 7.73) | 9.82  (8.75, 10.66) |
| Increase IAPV | 53.45% | 102.75% | 23.00% | 20.92% | 113.11% | 29.21% | 19.28% | 49.90% | 47.28% |
| Baseline^1^ tidalvolume^2^ | 5.68  (5.66, 5.74) | 4.62  (4.34, 4.85) | 6.48  (6.44, 6.53) | 8.29  (8.16, 8.44) | 5.80  (5.46, 6.03) | 6.47  (6.14, 6.94) | 5.55  (5.46, 5.66) | 4.77  (4.73, 4.85) | 6.94  (6.90, 6.96) |
| ERCC^1^ tidalvolume^2^ | 7.78  (7.50, 8.15) | 6.65  (6.55, 6.85) | 8.69  (8.41, 8.91) | 14.38  (13.70, 14.46) | 11.45  (11.07, 11.92) | 7.98  (7.05, 8.38) | 7.49  (7.36, 7.63) | 7.15  (6.79, 7.41) | 8.70  (8.28, 9.15) |
| Increase ERCC | 36.79% | 43.72% | 34.10% | 73.46% | 97.41% | 23.35% | 34.95% | 49.90% | 25.39% |
| PEEP [mBar] | 20 | 15 | 14 | 19 | 14 | 13 | 11 | 13 | 9 |
| Driving pressure [mBar] | 15 | 15 | 15 | 12 | 14 | 21 | 18 | 9 | 15 |
| ^1^Median (IQR), ^2^Tidalvolumes expressed in ml/kgPBW, | | | | | | | | | |

**S1 Table. Tidal volumes of individual patients before and during IAPV/ERCC, PEEP and Driving pressure values.** This table details individual patient tidal volumes and pressure settings pre- and during intervention.
