# Supplementary material for: Active expiration reduces hypercapnia in lung failure – results of the prospective interventional ActiveEx study and development of a prototype device for automated application
Source: PLoS One. 2025 Oct 16;20(10):e0333579. doi: 10.1371/journal.pone.0333579 (PMC12530571; doi:10.1371/journal.pone.0333579)
Supplement: S2 Table — This table summarizes secondary outcomes recorded during the study. (DOCX) [file pone.0333579.s005.docx]

**S2 Table.** **Further outcomes.** This table summarizes secondary outcomes recorded during the study.

|  | **IAPV,** N = 9^1^ | | **ERCC,** N = 9^1^ | | | | | |
| --- | --- | --- | --- | --- | --- | --- | --- | --- |
| **Characteristic** | **baseline** | **During / *Following*** | |  | **Baseline** | **During / Following** |  |  |
| *Tidal volume before and after IAPV/ERCC [mL]* | 439 (400, 495) | *440 (389, 522)* | |  | 392 (376, 522) | *448 (360, 495)* |  |  |
| *Arterial oxygen saturation after IAPV/ERCC [%]* | 95.1 (94.7, 95.3) | *93.2 (86.5, 95.2)* | |  | 95.70 (94.33, 96.73) | *92.70 (91.80, 94.10)* |  |  |
| Missing values |  |  | |  | 1 | 1 |  |  |
| Resistance during IAPV/ERCC (kpa/L/s) | 8.00 (7.68, 12.09) | 9.26 (6.79, 12.17) | |  | 8.51 (7.03, 10.52) | 8.87 (6.80, 11.24) |  |  |
| Missing values |  |  | |  | 1 | 2 |  |  |
| Peak inspiratory pressure during IAPV/ERCC [mbar] | 33 (31, 41) | 34 (29, 38) | |  | 37 (33, 39) | 36 (33, 39) |  |  |
| Missing values |  |  | |  | 1 | 1 |  |  |
| *Intrinsic PEEP after IAPV/ERCC [mbar]* | 14.9 (13.0, 18.1) | *14.0 (13.0, 16.0)* | |  | 15.00 (14.15, 17.40) | *15.10 (14.35, 19.50)* |  |  |
| Missing values | 1 | 1 | |  | 2 | 2 |  |  |
| *Tracheobronchial secretions [mg]* | 0.65 (0.64, 0.86) | *0.60 (0.58, 0.72)* | |  | 0.65 (0.62, 0.72) | *0.67 (0.65, 0.78)* |  |  |
| Missing values | 1 | 1 | |  | 1 | 1 |  |  |
| Central venous pressure during IAPV/ERCC [mmHg] | 11.0 (9.5, 15.0) | 11.4 (9.1, 14.2) | |  | 10.0 (9.0, 12.0) | 11.8 (8.6, 14.2) |  |  |
| Missing values | 2 | 2 | |  | 2 | 2 |  |  |
| PaO2/FiO2 | 138 (133, 146) |  | |  |  | 141 (139, 172) ^2^ |  |  |
| ^1^n (%); Median (IQR), ^2^within three hours after the intervention | | | | | | | | |
