# Supplementary material for: Active expiration reduces hypercapnia in lung failure – results of the prospective interventional ActiveEx study and development of a prototype device for automated application
Source: PLoS One. 2025 Oct 16;20(10):e0333579. doi: 10.1371/journal.pone.0333579 (PMC12530571; doi:10.1371/journal.pone.0333579)
Supplement: S3 Table — This table compares tidal volume data under baseline and automated compression conditions (laboratory simulations on a dummy). (DOCX) [file pone.0333579.s006.docx]

**S3 Table.** **Tidal volumes without and during automated compression (ERCC, IAPV).** This table compares tidal volume data under baseline and automated compression conditions (laboratory simulations on a dummy).
