# Supplementary material for: Active expiration reduces hypercapnia in lung failure – results of the prospective interventional ActiveEx study and development of a prototype device for automated application
Source: PLoS One. 2025 Oct 16;20(10):e0333579. doi: 10.1371/journal.pone.0333579 (PMC12530571; doi:10.1371/journal.pone.0333579)
Supplement: S1 File — (DOCX) [file pone.0333579.s007.docx]

**Titel der Studie**
Die AktivEx Studie – Aktive manuelle Unterstützung der Exspiration bei invasiv beatmeten Patient*innen

**Intervention:**
Ausübung intermittierender, leichter, händischer Kompressionen des Oberbauchs und unteren Thoraxs jeweils dann, wenn die Exspiration stattfindet oder in Atempausen zur Erzeugung eines Pinsp-unabhängigen Atemzugs. Es soll über 20 Minuten IAPV und nach 20-minütiger Pause ERCC durchgeführt werden.

**Interventionszeitraum:**
Einmalig 20 Minuten IAPV und anschließend nach 20-minütiger Pause einmalig 20 Minuten ERCC

**Primäre Hypothese:**
Durch IAPV/ERCC während der Exspiration oder in der Atempause können bei invasiv-beatmeten Patient*innen In- und Exspirationsvolumina vergrößert und zusätzliche, effektive Atemzüge generiert werden, ohne Erhöhung bzw. Applikation des Pinsp.
Die durch die Intervention vergrößerten bzw. generierten In- und Exspirationsvolumina führen zur Abnahme des pCO₂.

**Sekundäre Hypothesen:**

- Durch die Intervention kommt es zur Entblähung der Lunge und zur Wiedereröffnung von Atelektasen.
- Beide Effekte führen zu einer nachhaltig gebesserten Lungenfunktion.

**Primärer Endpunkt:**
**Effektivität:**

- In- und Exspirationsvolumina vor, während und nach Intervention.
- pCO₂ (Kapnographie/Blutgasanalyse) vor, während und nach Intervention.

**Sekundäre Endpunkte:**
**Respiration:**

- Blutgasanalyse vor und nach IAPV/ERCC
- Dynamische und statische Compliance vor, während und nach IAPV/ERCC
- Resistance vor, während und nach IAPV/ERCC
- Exspirationsflow
- Atemwegsspitzendruck vor, während und nach IAPV/ERCC
- Druck/Volumen Flusskurven Analyse vor und nach IAPV/ERCC
- In- und extrinsischer PEEP vor, während und nach IAPV/ERCC

*(Die Blutgasanalyse erfolgt in einer kleinen arteriellen Blutprobe, die über einen ohnehin bei den Patient*innen liegenden arteriellen Katheter entnommen wird. Die Messung der respiratorischen Parameter erfolgt nicht-invasiv über das Beatmungsgerät.)*

**Hämodynamik:**

- Herzfrequenz Baseline, während des IAPV/ERCC und am Ende. Dokumentation von etwaigen Rhythmusauffälligkeiten
- Blutdruckanalyse (systolisch/diastolisch/MAD) vor, während und nach IAPV/ERCC
- Zentraler Venendruck vor, während und nach IAPV/ERCC

*(Die Messungen erfolgen im Rahmen des bei beatmeten Patient*innen üblichen hämodynamischen Monitorings.)*

**Atelektasen:**

- Röntgenaufnahmen (ausschließlich, falls im Rahmen der Routinetherapie angefertigt)

**Kompressionsdruck:**

- Verblindete Messung des händisch applizierten Drucks mittels Flächendrucksensor

**Beobachtungszeitraum:**
Der primäre und sekundäre Endpunkt wird ab 48 Stunden vor, während und bis 48 Stunden nach Intervention beobachtet.

**Umfang und Aufbau des medizinisch-wissenschaftlichen Vorhabens:**
Pilotstudie mit 20 Patient*innen ohne Kontrollgruppe

**Einschlusskriterien:**

- Alter >18 Jahre
- Intensivmedizinische Therapie mit invasiver mechanischer Beatmung jeglicher Indikation
- Hyperkapnie (pCO₂ ≥ 45 mmHg)
- PEEP ≥ 8 mbar

**Ausschlusskriterien:**

- Pneumothorax
- Frakturen, Zustand nach Traumata und Verletzungen
- Jegliche Art von einliegenden Drainagen (insbesondere Thorax- und abdominelle Drainagen)
- Extrakorporales Lungenersatzverfahren
- Intraabdominelle Infektion
- Relevante Wunden an Thorax oder Abdomen
- Intraabdominelle, thorakale, gastrointestinale und/oder intrazerebrale Blutungen
- Hämophilie/schwere Thrombozytopenie (<20)
- Akutes Leberversagen (Bilirubin > 8 und Quick < 50)
- Schwere Kreislaufinstabilität
- Schwangerschaft

**Abbruchkriterien**

- Patientenwunsch
- Abfall der Sauerstoffsättigung und/oder des Blutdrucks (<90%, <60 mmHg MAP)
- Paradoxe Verringerung des Atemzugvolumens
- Ausdruck von Schmerz oder Angst
- Relevante Hypertonie
- Relevante Herzrhythmusstörungen

**Biometrische Planung und statistische Methodik**

- Bei der Studie handelt es sich um eine Pilotstudie ohne Kontrollgruppe mit explorativem Charakter, weshalb auf eine Fallzahlkalkulation verzichtet wird.
