# Supplementary material for: Active expiration reduces hypercapnia in lung failure – results of the prospective interventional ActiveEx study and development of a prototype device for automated application
Source: PLoS One. 2025 Oct 16;20(10):e0333579. doi: 10.1371/journal.pone.0333579 (PMC12530571; doi:10.1371/journal.pone.0333579)
Supplement: S2 File — (DOCX) [file pone.0333579.s008.docx]

**The AktivEx Study – Active Manual Support of Expiration in Invasively Ventilated Patients**

**Intervention**

**Procedure:**
Intermittent, gentle, manual compressions of the upper abdomen and lower thorax are applied during expiration or in breathing pauses to create a P_insp-independent breath. The intervention involves 20 minutes of IAPV followed by a 20-minute pause and then 20 minutes of ERCC.

**Intervention Period:**

- One-time 20 minutes of IAPV.
- After a 20-minute pause, one-time 20 minutes of ERCC.

**Primary Hypothesis**

IAPV and ERCC applied during expiration or in breathing pauses can increase inspiratory and expiratory volumes and generate additional, effective breaths without an increase in or application of P_insp.
The increased inspiratory and expiratory volumes induced by the intervention lead to a reduction in pCO₂.

**Secondary Hypotheses**

- The intervention leads to lung deflation and reopening of atelectasis.
- Both effects result in a sustained improvement in lung function.

**Primary Endpoint**

**Effectiveness:**

- Inspiratory and expiratory volumes before, during, and after the intervention.
- pCO₂ (via capnography and blood gas analysis) before, during, and after the intervention.

**Secondary Endpoints**

**Respiration:**

- Blood gas analysis before and after IAPV/ERCC.
- Dynamic and static compliance before, during, and after IAPV/ERCC.
- Resistance before, during, and after IAPV/ERCC.
- Expiratory flow before, during, and after IAPV/ERCC.
- Airway peak pressure before, during, and after IAPV/ERCC.
- Pressure/volume flow curve analysis before and after IAPV/ERCC.
- Intrinsic and extrinsic PEEP before, during, and after IAPV/ERCC.

*Note:* Blood gas analysis will be performed using small arterial samples obtained from pre-existing arterial catheters. Respiratory parameters will be measured non-invasively using the ventilator.

**Hemodynamics:**

- Heart rate at baseline, during IAPV/ERCC, and at the end, with documentation of any rhythm abnormalities.
- Blood pressure analysis (systolic, diastolic, MAP) before, during, and after IAPV/ERCC.
- Central venous pressure before, during, and after IAPV/ERCC.

*Note:* These measurements will be conducted as part of the standard hemodynamic monitoring for ventilated patients.

**Atelectasis:**

- Chest X-rays (only if performed as part of routine therapy).

**Compression Pressure:**

- Blinded measurement of manually applied pressure using surface pressure sensors.

**Observation Period**

Primary and secondary endpoints will be observed from 48 hours before, during, and up to 48 hours after the intervention.

**Scope and Design of the Study**

**Study Type:**

- Pilot study with 20 patients and no control group.

**Inclusion Criteria**

- Age >18 years.
- Intensive care treatment with invasive mechanical ventilation for any indication.
- Hypercapnia (pCO₂ ≥ 45 mmHg).
- PEEP ≥ 8 mbar.

**Exclusion Criteria**

- Pneumothorax.
- Fractures, recent trauma, or injuries.
- Any type of indwelling drains (especially thoracic and abdominal drains).
- Extracorporeal lung support.
- Intra-abdominal infection.
- Significant chest or abdominal wounds.
- Intra-abdominal, thoracic, gastrointestinal, or intracerebral bleeding.
- Hemophilia or severe thrombocytopenia (<20).
- Acute liver failure (bilirubin > 8 and Quick < 50).
- Severe hemodynamic instability.
- Pregnancy.

**Termination Criteria**

- Patient withdrawal
- Drop in oxygen saturation and/or blood pressure (<90%, <60 mmHg MAP)
- Paradoxical decrease in tidal volume
- Expression of pain or anxiety
- Significant hypertension
- Relevant cardiac arrhythmias

**Biometric Planning and Statistical Methodology**

- This study is a pilot study without a control group and is exploratory in nature; therefore, no sample size calculation is performed.
