# Supplementary material for: Active expiration reduces hypercapnia in lung failure – results of the prospective interventional ActiveEx study and development of a prototype device for automated application
Source: PLoS One. 2025 Oct 16;20(10):e0333579. doi: 10.1371/journal.pone.0333579 (PMC12530571; doi:10.1371/journal.pone.0333579)
Supplement: Attachment — Submitted filename: Response to Reviewers.docx [file pone.0333579.s011.docx]

Dear Dr. Chenette,

Dear Academic Editors and Reviewers,

We are grateful for the opportunity to revise and resubmit our manuscript to PLOS ONE. We sincerely thank the academic editor and reviewer for their insightful comments and helpful suggestions. We have carefully addressed each of the points raised and made appropriate changes to the manuscript as required. All changes made to the manuscript have been marked in blue font for clarity. Below, we provide a point-by-point response to all the comments.

We firmly believe that the insightful comments we received have substantially augmented the quality of our manuscript. Once again, we extend our deepest thanks for your expert guidance and unwavering support.

Sincerely,

The Authors

# Responses to Editorial Comments

**1. Please ensure that your manuscript meets PLOS ONE's style requirements, including those for file naming.**

We have carefully reviewed the PLOS ONE formatting guidelines and ensured that our manuscript adheres to all specified style requirements. This includes the use of appropriate section headings, formatting of references, figure citations, and file naming conventions. All elements of the manuscript now conform to the standards outlined in the official PLOS ONE style templates.

**Changes to the Manuscript:**

To ensure full compliance with PLOS ONE’s style requirements, the following formatting and structural revisions were made to the manuscript:

- **Title page:** The formatting of the title page, author byline, and affiliations has been adjusted according to the PLOS ONE guidelines. Symbols for equal contribution, current adress, and corresponding author have been applied as instructed.
- **Headings:** All Level 1,Level 2 and Level 3 headings were reformatted to match PLOS ONE’s style guidelines.
- **Figure and Table Citations:** All in-text citations for figures and tables were reviewed and corrected to ensure accurate placement and numbering.
- **Figure Captions:** Each figure caption was placed directly after the paragraph in which the figure is first cited, as required by journal guidelines.
- Figures: The order of Figure 4 (A, B, and C) has been changed to match their correct sequence as they appear in the manuscript.
- **Table Placement:** All tables were included immediately following the paragraph in which they are first cited in the main text.
- **References:**
  - The reference list was positioned after the main text and before the Supporting Information section.
  - All references were formatted in square brackets.
  - For references with more than six authors, only the first six names are listed, followed by “et al.” in accordance with PLOS ONE’s referencing style.
  - The references have been formatted according to journal standards, indicating the volume number, issue number in parentheses, and the page range or article ID of the publication.
- **Supporting Information Captions:** Captions for all Supporting Information files were listed at the end of the manuscript in a dedicated section titled **“Supporting Information”**, as per journal requirements.
- Supporting Information: The S Figures and S Tables are now provided as separate files with the corresponding formatting of the supporting information captions.
- Information on funding and competing interests has been removed from the Acknowledgments section.

**2. Please upload a new copy of Figure 5 as the detail is not clear.**

We thank the reviewer for the helpful comment regarding Figure 5. In the revised version, we have improved the graphical design to enhance clarity and readability in line with the journal’s figure preparation guidelines. The previously included percentage values have been removed, as they may have led to misinterpretation. Additionally, the figure legend and accompanying text have been revised for greater precision and to better reflect the experimental conditions. Importantly, the former bar plots have been replaced with box plots, as these not only display group-wise trends but also provide additional information on data dispersion and variability.

**Changes to the Manuscript:**

**Figure 5:** ~~Tidalvolumes of automated chest compression in a simulated intensive care environment: Figure A displays the average tidal volume over periods of 20 minutes, with automated IAPV at variaed PEEP but constant driving pressure.It shows the percentage increase in tidal volumes. Figure B presents the average tidal volumes measured over 20-minute intervals, both without expiratory assistance and with automated IAPV at PEEP levels.~~ **Effect of automated chest compression on tidal volumes in a simulated intensive care environment: Comparison of tidal volumes over 120 mechanically delivered breaths at PEEP levels of 5, 10, and 15 mBar, without (Baseline) and with automated inspiratory assistance (IAPV and ERCC), under constant driving pressure (5 mBar).**

(page 12, line 307ff)

**3. Please include captions for your Supporting Information files at the end of your manuscript, and update any in-text citations to match accordingly.**

We thank the editor for this note. All Supporting Information files have been updated accordingly, and the corresponding captions have been added at the end of the manuscript. In-text citations have been revised to ensure consistency and correct referencing throughout the text.

**Changes to the Manuscript:**

**Supporting information**

**S1 Fig.** **Flowchart on data collection and execution of the study.** This figure illustrates the process of data collection and the execution timeline and methodology of the study.
**S2 Fig. Air-filled cushion used for manual pressure measurement.** Self-constructed manual pressure sensor used during IAPV (one sensor) or ERCC (two sensors)
**S3 Fig.** **Visualization of pulmonary ventilation by PulmoVista.** This figure presents images of lung ventilation patterns as recorded by the PulmoVista system.

**S1 Table.** **Tidal volumes of individual patients before and during IAPV/ERCC, PEEP and Driving pressure values.** This table details individual patient tidal volumes and pressure settings pre- and during intervention.
**S2 Table.** **Further outcomes.** This table summarizes secondary outcomes recorded during the study.
**S3 Table. Tidal volumes without and during automated compression (ERCC, IAPV).** This table compares tidal volume data under baseline and automated compression conditions (laboratory simulations on a dummy).

(page 17, line 491ff)

**Responses to Reviewer Comments**

**1. Is the manuscript technically sound, and do the data support the conclusions?

The manuscript must describe a technically sound piece of scientific research with data that supports the conclusions. Experiments must have been conducted rigorously, with appropriate controls, replication, and sample sizes. The conclusions must be drawn appropriately based on the data presented.**

**Reviewer #1: No**

We thank the reviewer for this important and fundamental comment. We acknowledge that the study's single-arm design, small sample size, and focus on short-term physiological responses limit the ability to draw generalized or causal conclusions. However, we would like to emphasize that this work was explicitly conceptualized and conducted as a feasibility and pilot study, aiming to explore the short-term effects and safety of IAPV and ERCC in critically ill, hypercapnic patients, as well as to assess the technical feasibility of automating these interventions in a simulated ICU setting.

To clarify this intention, we have revised the Abstract, Introduction and Discussion sections of the manuscript to more explicitly highlight the exploratory nature and limitations of our design. We have removed the paragraph comparing IAPV and ERCC to ECMO, as we agree that the reference was speculative and not sufficiently supported by the scope and data of this exploratory study. We acknowledge that ECMO represents a fundamentally different intervention, and drawing direct parallels may be misleading at this stage.

We have revised the paragraph discussing diaphragmatic motion to avoid overinterpretation. While a trend toward increased diaphragmatic excursion was observed, we now present this as a hypothesis-generating finding, and acknowledge that diaphragm dysfunction is a known contributor to weaning failure which warrants further investigation in this context.

Moreover, we have adjusted our conclusions to avoid overgeneralization and to better reflect the scope and constraints of our study.

Changes to the manuscript:

Section “Abstract”

Manual and automated ventilator-synchronized IAPV and ERCC ~~significantly improved ventilation~~ were associated with improved ventilation. ~~These techniques hold promise for standardized use in hypercapnic conditions, including weaning failure, obstructive respiratory diseases, poor lung compliance, and muscular respiratory weakness.~~ Their potential role in managing hypercapnic respiratory failure—such as in weaning failure, obstructive lung disease, or neuromuscular weakness—remains a subject for future clinical research.

(page 1f, line 63ff)

Section “Introduction”

Given the lack of robust clinical data on IAPV and ERCC in acute hypercapnic respiratory failure, this study was intentionally designed as a pilot and feasibility trial. Its primary goal was to generate preliminary physiological insights, assess technical safety, and explore the potential for automated application in a controlled setting. It was not powered nor designed to evaluate long-term outcomes or to support confirmatory conclusions.

(page 4, line 102ff)

Section “Discussion”

~~ECMO (extracorporeal membrane oxygenation) is the logical intervention as it provides sufficient CO2 removal and oxygenation support when conventional ventilation strategies fail. However, ECMO therapy, especially in elderly patients, may not be suitable for everyone. In contrast, IAPV and ERCC are minimally invasive techniques,can be implemented quickly, and are feasible also in smaller hospitals. IAPV and ERCC offer a valuable alternative by enhancing ventilation and potentially stabilizing patients until ECMO can be initiated, or in cases where ECMO is not immediately available or contra indicated.~~

(page 12, line 327ff)

We observed a trend toward increased diaphragmatic movement during IAPV and ERCC. Since diaphragm dysfunction and atrophy frequently contribute to weaning failure in mechanically ventilated patients [16], such techniques may help engage the diaphragm. Whether this effect could support weaning processes remains a hypothesis to be tested in future studies.

~~We noted increased diaphragmatic movement during IAPV and ERCC. As the diaphragm rapidly suffers from hypotrophy under invasive ventilation the concept of diaphragmatic support by IAPV or ERCC might be implemented in weaning therapy [16].~~

(page 13, line 344ff)

This study has several important limitations. It was designed as a single-arm, non-randomized pilot study, with a small sample size and a short observation period, ~~The study was conducted at a single center with a small sample size~~ which ~~may~~ limit the generalizability of the results.

(page 14, line 373f)

It was designed as a single-arm, non-randomized pilot study, with a small sample size and a short observation period. The lack of a control group restricts any causal inferences. While the statistical analysis revealed significant effects on tidal volumes and partial pressure of CO₂, several secondary outcomes, such as diaphragmatic motion, showed non-significant trends only. Future studies should include larger, controlled patient cohorts and evaluate the effects of these techniques on long-term outcomes, such as weaning success or ICU length of stay.

(page 14, line 380ff)

It should be emphasized that the automated device was tested exclusively in a simulated ICU environment, which limits the applicability of these findings to real-life clinical settings.

(page 15, line 398f)

Section “Conclussion”

~~The study demonstrates that both IAPV and ERCC are effective, fast, and easy-to-implement techniques for reducing hypercapnia in patients with lung failure. Both manual and automated approaches significantly increased tidal volumes. The automated prototype device for IAPV and ERCC, synchronized with the ventilator, proved to be as effective as manual application, offering significant potential for standardized and automated use in clinical practice. Furthermore, synchronization with the ventilator opens possibilities for personalized ventilation strategies, allowing adjustments based on individual patient needs.~~

This exploratory study suggests that both IAPV and ERCC may be feasible and rapidly applicable techniques to enhance ventilation and reduce hypercapnia in patients with hypercapnic respiratory failure. In our limited sample, both manual and automated approaches were associated with increased tidal volumes. The automated prototype device, synchronized with the ventilator, demonstrated comparable effects to manual application under simulated conditions, indicating potential for standardized and automated use pending further clinical validation. Moreover, ventilator-synchronized compression may allow future adaptation to individual respiratory patterns, supporting the concept of personalized assistance strategies.

However, given the pilot nature of this study and the preclinical character of the automated prototype testing, further clinical studies are required to evaluate the efficacy, tolerability, and long-term safety of these interventions in broader patient populations.

(page 15f, line 413ff)

**2. Has the statistical analysis been performed appropriately and rigorously?**

**Reviewer #1: No**

We appreciate this critical comment and have taken it very seriously. In response, we have fundamentally revised the statistical analysis to ensure methodological rigor and appropriateness. The revised analysis was conducted in close collaboration with the Institute of Biometry and Clinical Epidemiology, Charité – Universitätsmedizin Berlin. In this context, we have also revised Figures 3 and 5 to reflect the updated results and improve the clarity and informativeness of the visual presentation.

Specifically, we replaced the Brunner-Munzel test with the paired-sample nonparametric t-test based on relative effects, which is specifically designed for dependent (paired) observations. All relevant analyses and sections of the manuscript have been revised accordingly to reflect this change.

Revision Figure 3A and 3C: The previously presented boxplots of tidal volumes combined all measurement time points without distinguishing between individual subjects. As a result, the within-subject dependency was ignored, and the data were incorrectly visualized as independent. In the revised figure, the medians of the tidal volumes per person before and during the intervention are now presented to avoid within-subject dependency.

Revision Figure 5 (Effect of automated chest compression on tidal volumes in a simulated intensive care environment): The former bar plots have been replaced with box plots, as these not only display group-wise trends but also provide additional information on data dispersion and variability.

All corresponding changes to the manuscript are detailed in Section 5.4.

**3. Have the authors made all data underlying the findings in their manuscript fully available?

The PLOS Data policy requires authors to make all data underlying the findings described in their manuscript fully available without restriction, with rare exception (please refer to the Data Availability Statement in the manuscript PDF file). The data should be provided as part of the manuscript or its supporting information, or deposited to a public repository. For example, in addition to summary statistics, the data points behind means, medians and variance measures should be available. If there are restrictions on publicly sharing data—e.g. participant privacy or use of data from a third party—those must be specified.**

**Reviewer #1: Yes**

Thank you for confirming full compliance with the PLOS Data policy.

**4. Is the manuscript presented in an intelligible fashion and written in standard English?

PLOS ONE does not copyedit accepted manuscripts, so the language in submitted articles must be clear, correct, and unambiguous. Any typographical or grammatical errors should be corrected at revision, so please note any specific errors here.**

**Reviewer #1: Yes**

We appreciate the reviewer’s positive comments on the clarity and language quality.

**5. Review Comments to the Author**

**5.1 The manuscript and the study protocol indicate that because this was deemed a pilot study, no sample size calculations were conducted. However, the protocol calls for 20 patients. However only 10 were enrolled, one withdrew, and nine were analyzed. There is no explanation for stopping enrollment after 10 patients.**

Thank you for this important observation. We have added a detailed explanation in the revised manuscript. As outlined, this was an exploratory pilot study with a planned sample size of 20 patients. However, recruitment was stopped after 10 patients because the observed effects were consistent and pronounced, and further inclusion of patients was not deemed to add relevant clinical or methodological value within the single-arm design. This decision was made in accordance with the study’s exploratory nature and without inferential hypothesis testing. We have now clarified this rationale in the Methods and Results sections of the manuscript.

Changes to the manuscript:

Section “Methods”

This study was designed as an exploratory, single-arm pilot trial with a planned enrollment of 20 patients. No formal sample size calculation was performed, consistent with the study’s exploratory nature. During the course of the study, recruitment was stopped after 10 patients due to the consistent and pronounced treatment effects observed in this group. As no control group was included and further enrollment was not expected to substantially change the overall interpretation or add significant insights, the decision was made to discontinue recruitment. This decision was made before any formal analysis and without conducting any statistical hypothesis testing.

(page 5, line 119ff)

Section “Results”

Recruitment was stopped after these 10 patients due to the consistent and marked effects observed, which were considered sufficient to meet the exploratory aims of the study.

(page 6, line 174ff)

**5.2 All patients were treated first with IAPV for twenty minutes, rested for 20 minutes, and then treated with ERCC. It would have been preferable to randomize the order in which the patients received the treatments.**

We thank the reviewer for this important methodological comment. We fully agree that randomizing the order of interventions would have been preferable to avoid potential sequence or carryover effects. In this initial feasibility study, we deliberately chose a fixed sequence (IAPV followed by ERCC) to minimize logistical complexity and ensure procedural consistency across the small cohort. However, we acknowledge that this limits the interpretability of comparative findings, and we now explicitly state this limitation in the manuscript. Future studies should incorporate randomization of intervention order to allow more robust comparisons.

Changes to the manuscript:

Notably, the sequence of interventions was not randomized, with all patients receiving IAPV prior to ERCC. This fixed order may have introduced carryover or timing effects and limits the strength of direct comparative conclusions regarding clinical effectiveness.

(page 13, line 355ff)

**5.3 Reference number 12 lists the year incorrectly; it is The R Journal, 2023; 15(1):142-158.**

Thank you very much for the helpful remark. Reference number 12 is no longer included in the revised manuscript, as the corresponding test has been replaced (see 5.4).

**5.4 The most serious concern is that the statistical test used to compare the pre- and post- respiratory and hemodynamic outcome measures, the Brunner-Munzel test, is inappropriate for these data. This test is for data arising from two independent samples, but the data are collected before and after treatment on the same nine individuals, and thus are paired data, not from two independent samples.**

Thank you very much for this valuable comment. We fully agree that the Brunner-Munzel test is not appropriate for paired data, as it assumes independent samples. In response, we consulted the Institute of Biometry and Clinical Epidemiology of Charité. Based on their advice, we replaced the Brunner-Munzel test with the paired-sample nonparametric t-test based on relative effects, which is specifically designed for dependent (paired) observations. All relevant analyses and sections of the manuscript have been revised accordingly to reflect this change.

It was also noted that Figure 3: Increased ventilation and decarboxylation by IAPV and ERCC originally presented boxplots of tidal volumes that combined all measurement time points without distinguishing between individual subjects. As a result, the within-subject dependency was ignored, and the data were incorrectly visualized as independent. In the revised figure, the medians of the tidal volumes before and during the intervention are now presented.

The original Figure 5 (**Effect of automated chest compression on tidal volumes in a simulated intensive care environment.)** displayed only the average of the automated ventilation. The figure has now been replaced with a boxplot representation to also illustrate the measure of variability.

Changes to the manuscript:

Title page:

Mareen Pigorsch and the Institute of Biometry and Clinical Epidemiology, Charité – Universitätsmedizin Berlin (corporate member of Freie Universität Berlin and Humboldt-Universität zu Berlin), Berlin, Germany, has now been added to the author list to reflect their substantial contribution to the statistical analysis.

Abstract:

**In nine hypercapnic patients, manual IAPV increased tidal volume from 5.72 to 8.85 ~~mL/kg PBW~~ mL / kg predicted bodyweight (p<0.001, relative effect (CI) ~~0.89~~** ~~(0.84 – 0,94)~~ 0.93 (0.79 – 1.06)**) and ERCC from 5.79 to 8.13 ~~mL/kg PBW~~ mL / kg predicted bodyweight (p<0.001, relative effect (CI)** ~~0.90 (0.86 – 0.95)~~ 0.93 (0.80 – 1.05)**). PaCO2 significantly reduced after 20 minutes with both techniques (IAPV: from 65 to 52 mmHg, p<0.01, relative effect (CI) 0.15** ~~(-0.06 - 0.35)~~ (0.01 – 0.28)**; ERCC: from 61 to 51 mmHg, p=~~0.03~~ <0.01, relative effect (CI)** 0.22 ~~(-0.02 - 0.48)~~ (0.07 – 0.37)**).**

(page 2, line 54ff)

Section “Statistical analysis”

~~Brunner-Munzel (R package rankFD [12]) test for metric outcome comparison (pre- and post-study, suitable for non-normal, heterogeneous variance data) was used.~~ To compare pre- and post-intervention measurements, consisting of metric paired data, we applied a nonparametric paired-sample t-test based on relative effects (R package nparcomp [12]). ~~Significance threshold was set at p=0.05, comparing outcomes by a relative effect p-value.~~ The significance threshold was set at α = 0.05. Outcome comparisons were based on a nonparametric test of the relative effect (p), with corresponding p-values reported for statistical inference.

(page 6, line 158ff)

Section “Results”

While performing IAPV, ~~average~~ median tidal volumes expressed in mL per kg predicted body weight increased by 42%, i.e. from 6.23 (5.72, 6.86) mL / kg **predicted bodyweight** to 8.85 (7.73, 10.54) mL / kg **predicted bodyweight** (p=<0.001, **relative effect (CI) ~~0.89~~** ~~(0.84 – 0,94)~~ 0.93 (0.79 – 1.06)) (Fig 3A). ERCC also led to an increase from 5.79 (5.40, 6.83) mL / kg **predicted bodyweight** to 8.13 (7.27, 9.15) mL / kg **predicted bodyweight** (p=<0.001**, relative effect (CI)** 0.93 (0.80 – 1.05)), equivalent to a change of 40,4% (Fig 3C).

Consequently, 20 minutes of IAPV demonstrated a considerable decrease in p_a_CO_2_ (Fig 3B) as median baseline dropped from 65 mmHg (54, 66) to 52 mmHg (39, 52) (p=<0.01, **relative effect (CI) 0.15** (0.01 – 0.28)). Similarly, ERCC exhibited a substantial reduction in p_a_CO_2_, decreasing from 61 mmHg (52, 65) to 51 mmHg (40, 58) (p=~~0.03~~ <0.01**, relative effect (CI) 0.22** (0.07 – 0.37)) after 20 minutes (Fig~~ure~~ 3D).

(page 8, line 202ff)

To further investigate the mode of action of IAPV and ERCC, diaphragmatic movement, tracheobronchial secretion, AADO_2_, and intrinsic PEEP were assessed. Diaphragmatic motion increased from 1.10cm (0.93, 1.18) to 1.65cm (1.37, 2.04) (p=~~0.15~~ 0.03, **relative effect (CI) 0.76** (0.50 – 1.01)) during IAPV and from 1.14cm (0.85, 1.38) to 1.34cm (1.24, 1.46) (p=~~0.22~~ 0.10, **relative effect (CI) 0.71** (0.43 – 1.00)) during ERCC. We observed no effect on tracheobronchial secretions. Notably, seven out of eight patients exhibited no tracheobronchial secretions. Intrinsic PEEP remained constant

(page 8, line 220ff)

~~AADO2 levels increased numerically from a median value of 263 (IQR: 220, 356) before the intervention to 319 (IQR: 257, 377) afterwards, with a p-value of 0.37.~~ Following the IAPV intervention, AADO₂ increased from a median of 263 (IQR: 220–356) to 319 (IQR: 257–377) (p < 0.01), while ERCC rose from 278 (IQR: 225–339) to 307 (IQR: 252–360) (p < 0.001).

(page 8, line 225ff)

**Figure 3:** Increased ventilation and decarboxylation by IAPV and ERCC. A) Depicts median tidal volumes before and during IAPV. B) p_a_CO_2_ values of individual patients before and after IAPV C) Illustrates median tidal volumes before and during ERCC. D) p_a_CO_2_ values of individual patients before and after ERCC are shown.

**Figure 5:** ~~Tidalvolumes of automated chest compression in a simulated intensive care environment: Figure A displays the average tidal volume over periods of 20 minutes, with automated IAPV at variaed PEEP but constant driving pressure.It shows the percentage increase in tidal volumes. Figure B presents the average tidal volumes measured over 20-minute intervals, both without expiratory assistance and with automated IAPV at PEEP levels.~~ **Effect of automated chest compression on tidal volumes in a simulated intensive care environment: Comparison of tidal volumes over 120 mechanically delivered breaths at PEEP levels of 5, 10, and 15 mBar, without (Baseline) and with automated inspiratory assistance (IAPV and ERCC), under constant driving pressure (5 mBar).**

**Revised Table 2: Respiratory and hemodynamic outcome parameters**

|  | **IAPV** N = 9 | | | |
| --- | --- | --- | --- | --- |
|  | **Before^1^** | **During/ *Following^1^*** | **p-value^2^** | **Relative effect^3^ (CI) ^4^** |
|  |  |  |  |  |
| Tidal volume**^5^** [mL / kg] | 6.23 (5.72, 6.86) | 8.85 (7.73, 10.54) | <0.001 | **~~0.89~~** ~~(0.84 – 0,94)~~ 0.93 (0.79 – 1.06) |
| pH value | 7.35 (7.24, 7.38) | *7.43 (7.38, 7.48)* | ~~0.02~~ <0.001 | 0.80 ~~(0.56 - 1.04)~~ (0.68 – 0.92) |
| p_a_CO_2_ [mmHg] | 65 (54, 66) | *52 (39, 52)* | <0.01 | 0.15 ~~(-0.06 - 0.35)~~ (0.01 – 0.28) |
| P_a_O_2_ [mmHg] | 82 (77, 92) | *64 (51, 81)* | 0.02 | 0.21 ~~(-0.04 – 0.46)~~ (-0.01 – 0.43) |
| P_a_O_2_/FIO_2_ | 140 (134, 146) | *102 (80, 116)* | ~~0.01~~ 0.02 | 0.12 (-0.13 – 0.38) |
| AADO_2_ | 263 (220, 356) | *319 (257, 377)* | ~~0.37~~ <0.01 | ~~0.62 (0.33 - 0.0.93)~~ 0.63 (0.57 – 0.69) |
| Compliance [ml/cm H_2_O] | 33 (23, 39) | 47 (33, 67) | ~~0.04~~ <0.001 | ~~0.78 (0.52 – 1.01)~~ 0.77 (0.68 – 0.86) |
| Diaphragmatic movement**^6^** [cm] | 1.10 (0.93, 1.18) | 1.65 (1.37, 2.04) | ~~0.15~~ 0.03 | 0.76 ~~(0.40 - 1.11)~~ (0.5 – 1.01) |
| Heartrate [bpm] | 86 (77, 97) | 83 (70, 97) | ~~0.71~~ 0.07 | 0.44 ~~(0.14 - 0.75)~~ (0.39 – 0.50) |
| MAP [mmHg] | 78 (76, 78) | 82 (80, 86) | <0.01 | 0.85 (0.63 – 1.07) |
| Manual applied pressure**^7^** [mbar] |  | *51 (41, 59)* |  |  |
|  |  |  |  |  |
|  | **ERCC** N = 9 | | | |
|  |  |  |  |  |
| Tidal volume**^5^** [mL / kg] | 5.79 (5.40, 6.83) | 8.13 (7.27, 9.15) | <0.001 | ~~0.90 (0.86 – 0.95)~~ 0.93 (0.80 – 1.05) |
| pH value | 7.35 (7.28, 7.38) | *7.46 (7.38, 7.49)* | ~~0.06~~ <0.01 | ~~0.84 (0.48 - 1.05)~~ 0.77 (0.56 – 0.95) |
| p_a_CO_2_ [mmHg] | 61 (52, 65) | *51 (40, 58)* | ~~0.03~~ <0.01 | 0.22 ~~(-0.02 - 0.48)~~ (0.07 – 0.37) |
| P_a_O_2_ [mmHg] | 95 (77, 104) | *69 (63, 82)* | ~~<0.01~~ 0.01 | 0.19 ~~(-0.03 - 0.40)~~ (0.00 – 0.37) |
| P_a_O_2_/FIO_2_ | 139 (131, 155) | *109 (102, 125)* | ~~0.02~~ 0.01 | 0.16 ~~(-0.02 - 0.42)~~ (-0.08 – 0.41) |
| AADO_2_ | 278 (225, 339) | *307 (252, 360)* | ~~0.48~~ <0.001 | ~~0.60 (0.30 - 0.91)~~ 0.61 (0.52 - 0.69) |
| Compliance [ml/cm H_2_O] | 23 (23, 38) | 35 (26, 49) | ~~0.14~~ <0.001 | 0.70 ~~(0.43 - 0.99)~~ (0.62 – 0.79) |
| Diaphragmatic movement**^6^** [cm] | 1.14 (0.85, 1.38) | 1.34 (1.24, 1.46) | ~~0.22~~ 0.10 | 0.71 ~~(0.34 - 1.08)~~ (0.43 – 1.00) |
| Heartrate [bpm] | 87 (77, 100) | 82 (78, 99) | ~~0.77~~ 0.10 | ~~0.46 (0.15 - 0.77)~~ 0.71 (0.43 – 1.00) |
| MAP[mmHg] | 76 (76, 85) | 86 (82, 88) | ~~0.21~~ 0.11 | 0.68 ~~(0.39 - 0.97)~~ (0.46 – 0.90) |
| Manual applied pressure**^7^** [mbar] |  | *84 (71, 94)* |  |  |
|  |  |  |  |  |

^1^Median (IQR); **^2^** paired-sample t-test; ^3^Relative effect p ~~(Brunner-Munzel test)~~; ^4^Confidence interval; Tidal volume expressed in mL per kg predicted body weight; **^6^**N=8 (one missing value); **^7^**N=X (3 missing values); p_a_CO_2_ = Partial Pressure of Carbon Dioxide in Arterial Blood; P_a_O_2_ = Partial Pressure of Oxygen in Arterial Blood, FIO_2_ = Fraction of Inspired Oxygen, AADO_2_ = Alveolar-arterial Oxygen Gradient, MAP = Mean Arterial Pressure
