# Supplementary material for: Active expiration reduces hypercapnia in lung failure – results of the prospective interventional ActiveEx study and development of a prototype device for automated application
Source: PLoS One. 2025 Oct 16;20(10):e0333579. doi: 10.1371/journal.pone.0333579 (PMC12530571; doi:10.1371/journal.pone.0333579)
Supplement: Attachment — Submitted filename: Response_to_Reviewers_auresp_2.docx [file pone.0333579.s012.docx]

**Response to Reviewer**

**We sincerely thank the reviewer for the careful re-evaluation of our revised manuscript and for the constructive minor comments. We very much appreciate the reviewer’s acknowledgment that the revised version has considerably improved, and we are grateful for the additional suggestions, which helped us to further enhance the clarity, precision, and overall quality of the manuscript. All comments have been carefully considered, and the requested corrections have been implemented accordingly. Our detailed responses are provided below, point by point:**

**1. If the authors have adequately addressed your comments raised in a previous round of review and you feel that this manuscript is now acceptable for publication, you may indicate that here to bypass the “Comments to the Author” section, enter your conflict of interest statement in the “Confidential to Editor” section, and submit your "Accept" recommendation.**

**Reviewer #1: (No Response)**

We note that no specific response was provided to this question. Nevertheless, we remain very grateful for the reviewer’s careful consideration of our work throughout the review process and for the constructive feedback offered in the other sections.

**2. Is the manuscript technically sound, and do the data support the conclusions?

The manuscript must describe a technically sound piece of scientific research with data that supports the conclusions. Experiments must have been conducted rigorously, with appropriate controls, replication, and sample sizes. The conclusions must be drawn appropriately based on the data presented.**

**Reviewer #1: Yes**

We appreciate the confirmation that the study is technically sound and that the data adequately support the conclusions. It is reassuring to know that the rigor of the experimental design and the interpretation of the results have been recognized.

**3. Has the statistical analysis been performed appropriately and rigorously?**

**Reviewer #1: Yes**

We are glad that the statistical analyses have been judged appropriate and rigorous. We invested significant effort to ensure robustness and transparency in this regard, and we appreciate the reviewer’s acknowledgement.

**4. Have the authors made all data underlying the findings in their manuscript fully available?

The** [**PLOS Data policy**](http://track.editorialmanager.com/CL0/http:%2F%2Fwww.plosone.org%2Fstatic%2Fpolicies.action%23sharing/1/010f01985a46b218-963c52e7-6a17-4e74-a263-a94f659986ce-000000/bj8vfIBImIB3Ag8TDOqC0RQs4rxzwjHv2rGBqcznUug=222) **requires authors to make all data underlying the findings described in their manuscript fully available without restriction, with rare exception (please refer to the Data Availability Statement in the manuscript PDF file). The data should be provided as part of the manuscript or its supporting information, or deposited to a public repository. For example, in addition to summary statistics, the data points behind means, medians and variance measures should be available. If there are restrictions on publicly sharing data—e.g. participant privacy or use of data from a third party—those must be specified.**

**Reviewer #1: Yes**

We thank the reviewer for confirming that the data underlying the findings are fully available. We made it a priority to follow the PLOS ONE data policy closely, and we are pleased that this has been recognized.

**5. Is the manuscript presented in an intelligible fashion and written in standard English?

PLOS ONE does not copyedit accepted manuscripts, so the language in submitted articles must be clear, correct, and unambiguous. Any typographical or grammatical errors should be corrected at revision, so please note any specific errors here.**

**Reviewer #1: Yes**

We are grateful for the reviewer’s comment that the manuscript is clearly written and intelligible. We carefully revised the text to improve clarity and readability, and we are glad these efforts were successful.

**6. Review Comments to the Author

Reviewer #1: This revised manuscript is considerably improved. I just have a few minor comments regarding the revision.**

**Overall, we would like to express our sincere gratitude to the reviewer for the constructive and positive evaluation of our work. The supportive comments are greatly appreciated, and we are encouraged by the recognition of the quality and clarity of our study.**

**R.1 line 150 - denoting relative effect by 'p' is confusing vis a vis p-values. please choose another symbol for relative effect.**

**We agree that the previous notation “p” for relative effect was confusing given its association with p-values. To avoid ambiguity, we have removed the symbol altogether. The results are now consistently reported as “relative effect” without using any additional symbol. We believe this wording is clearer and prevents potential misinterpretation by the reader.**

**R.2 Table 1: adjust column width so that the word 'ventilation' in the column heading is not split over two lines**

**The column width has been adjusted so that the term “ventilation” in the column heading is displayed on a single line.**

**R.3 line 205 - most measures are reported as median and IQR, but the manual compression pressure is reported as mean and IQR. Why switch from median to mean for this variable? Also, reporting IQR along with a mean is unusual. Generally one reports the standard deviation with means and IQR with medians.**

**We thank the reviewer for pointing this out. The indication of “mean” was a typographical error. Manual compression pressure was in fact analyzed and reported as median (IQR), consistent with the other variables. This has now been corrected in the text.**

**R.4 Typos - next to last sentence in Results section of Abstract 'rapid' should be 'rapidly'**

**- line 137 - need a space between 'pressure' and 'was'**

**- line237 'from' should be 'for'**

**There are a few places in the revised manuscript where the marked changes remain (ie, were not accepted or cleared, such as in line 248)**

**Abstract, Results section: corrected “rapid” → “rapidly”**

**Line 137: inserted missing space between “pressure” and “was”**

**Line 237: corrected “from” → “for”**

**All previously marked changes have now been accepted and cleared.**

**Off-topic correction – Age data in Table**

**To further ensure patient anonymity, the exact ages originally shown in Table 1 have been replaced by decade ranges (e.g., “43” → “40-50”). This modification has no impact on the results or interpretation but strengthens data protection.**

**Changes to the Manuscript**

**Line 109: To ensure patient confidentiality, exact ages were not reported; instead, all ages were categorized into decade ranges (e.g., 43 years → 40–50 years).**

**We are grateful for these helpful suggestions, which have further improved the clarity and consistency of our manuscript.**

**Sincerely,**

**The Authors**
